# Meeting report: 400 synthetic biology projects promote sustainable development

**DOI:** 10.1093/sumbio/qvaf018

**Published:** 2025-08-13

**Authors:** Diandra Daumiller, Carmen de Ancos Jimeno, Aleksandras Gaudiesius, Edna Hiu Tung Lam, Thi Huyen Mai Nguyen, Dimitrios Tsagkogiannis, Juliana Baranova, Felix Falk

**Affiliations:** Karolinska Institutet, 17177 Stockholm, Sweden; Department of Molecular Biosciences, Stockholm University, Science for Life Laboratory, 17165 Stockholm, Sweden; KTH Royal Institute of Technology, 10044 Stockholm, Sweden; Department of Protein Science, KTH Royal Institute of Technology, 10691 Stockholm, Sweden; Karolinska Institutet, 17177 Stockholm, Sweden; Karolinska Institutet, 17177 Stockholm, Sweden; Department of Protein Science, KTH Royal Institute of Technology, Science for Life Laboratory, 17165 Stockholm, Sweden; Department of Microbiology, Tumor and Cell Biology, Karolinska Institutet, Science for Life Laboratory, 17165 Stockholm, Sweden; Stockholm University, 10691 Stockholm, Sweden; Department of Physiology and Pharmacology, Karolinska Institutet, 17177 Stockholm, Sweden; Department of Cell and Molecular Biology, Uppsala University, 752 37 Uppsala, Sweden; Department of Medicine Huddinge, Karolinska Institutet, Center for Hematology and Regenerative Medicine Stockholm, 14152 Huddinge, Sweden

**Keywords:** Synthetic biology, Bioremediation, Metabolic engineering, In vitro models, Biological control of plant diseases

## Abstract

The 21st annual International Genetically Engineered Machine (iGEM) Grand Jamboree took place on the 23rd to 26th of October 2024 at the Paris Expo in Paris, France. It was the final event of the year-long iGEM Competition in microbial synthetic biology, at which more than four hundred high school and university student teams from around the world presented their projects and competed for prizes. At the core, each project applied molecular cloning and microbial engineering to tackle pressing healthcare, human advancement, and environmental challenges. Herein we highlight the efforts of four projects from the latter category: Stockholm, Thessaly, KU Leuven, and Copenhagen, whose work exemplifies the type of research projects presented by iGEM teams each year in advancing sustainability. Through the application of basic principles of synthetic biology to a diverse range of problems, the projects demonstrated the promise of synthetic biology in addressing and indeed resolving a broad spectrum of sustainability challenges.

**Sustainability Statement**

The iGEM projects presented herein directly involve several United Nations sustainable development goals, but are in their emphasis on sustainability and collaboration united in their efforts towards the 17th goal, *partnership for the goals*. Each project uses synthetic biology to address an issue related to sustainability, alongside community outreach efforts to raise awareness of the issue and learn about how it best can be tackled. The projects also involved issues related to goals 3, 6, 12, 14, and 16, pertaining to health, clean water, responsible production, and life below water and on land.

## Introduction

International Genetically Engineered Machine (iGEM) started in 2003 as a course at the Massachusetts Institute of Technology, where students designed synthetic DNA sequences to make cells blink using fluorescent signals. This has since developed into a nonprofit organization that advances synthetic biology through education, collaboration, and international competition (Jainarayanan et al. 2021). The leading principle is the application of synthetic biology and the engineering of biological systems to advance human and environmental prosperity (Cameron et al. 2014). Among iGEM’s efforts, the annual iGEM Competition is the most well-known (Jainarayanan et al. 2021). The iGEM Competition challenges student teams to create solutions to real-world problems using synthetic biology over the course of a year, and allows them to develop and execute their very own research project. A team will typically use the spring to brainstorm, plan, and design their project; the summer to perform it in the laboratory; and the autumn to summarize their work in the form of a website and presentation at the Jamboree. iGEM provides teams with essential resources, including a Distribution Kit with standardized genetic parts and access to DNA synthesis services through partnerships with companies like IDT and Twist Bioscience, while also facilitating collaborations between teams. At the end of October, every team is invited to the Grand Jamboree in Paris to present their project to peers and judges, compete for awards, and celebrate their achievements. To qualify for medals, a project must meet criteria centered around best practices in synthetic biology: documentation that future teams can build upon, adherence to the BioBrick genetic parts standard, demonstration of relevance of the solution through Human Practices activities, and reciprocity between design and implementation using the engineering cycle. Furthermore, teams excelling in specific areas such as educational activities, *in silico* modeling, documentation or inclusivity compete for special prizes (iGEM 2025).

Beyond the competition, iGEM aims to develop the field of synthetic biology on a global scale by, among other things, supporting future synthetic biology startups and providing arenas for discussion and collaboration (Jainarayanan et al. 2021). Their largest contribution to this end is arguably the BioBrick system, a modular DNA sequence standard that has allowed the establishment of a common library of DNA building blocks (Smolke 2009). Each BioBrick represents a functional genetic component such as a coding sequence, regulatory element or genetic marker. iGEM maintains the BioBrick system using the BioBrick Registry, allowing scientists and iGEM teams to order standardized genetic parts. To ensure the maintenance and growth of the Registry, each iGEM team must construct, utilize, or improve BioBricks as part of their project. This standardized library of well-characterized DNA parts is easy to navigate, convenient to use, and assemble into new designs in a sustainable way, therefore advancing research for efficient structure design and promoting collaboration.

The scope of projects has massively expanded since the start of the competition. In iGEM, student teams could, as of 2024, pick from 14 different villages, corresponding to different subject areas, to compete in. In addition to quintessential villages such as *diagnostics, biomanufacturing*, and *agriculture*, 2024 saw the addition of the *oncology, infectious diseases*, and *fashion and cosmetics* villages (iGEM 2025). Hence, iGEM encourages students to directly deepen and widen what is possible to do with synthetic biology. In line with this pursuit, the 2024 iGEM high school, undergraduate, and graduate Grand Prizes were awarded to the teams representing GEMS-Taiwan for their beetroot-inspired mosquito trap to combat dengue fever, Heidelberg for their CRISPR-based 3D genome engineering toolbox and Marburg for their dandelion BioBricks library for the production of natural rubber (GEMS-Taiwan - iGEM 2024 2025, Heidelberg - iGEM 2024 2025, Marburg - iGEM 2024 2025). To demonstrate the potential of iGEM teams in addressing sustainable development, we herein highlight the works of the Stockholm, Thessaly, KU Leuven, and Copenhagen teams. The projects utilized microbiology to address global challenges with a strong focus on sustainability and innovative approaches. We chose to highlight them because of their creative and engaging methods in tackling sustainability-related issues.

## Synthetic guanine crystals by iGEM Stockholm

The Stockholm overgraduate team competed in the *fashion and cosmetics* village with the project BioGlimmer, focusing on the largely unsustainable production of cosmetics ingredients. Responding to the growing demand for vegan, environmentally friendly, and ethically sourced cosmetics, the team aimed to produce the ubiquitous cosmetic ingredient shimmer using synthetic biology as an alternative to the current sources: mica, specific types of silicate minerals, and guanine crystals extracted from fish scales (Lebsack 2025). Inspired by the finding by Pavan and colleagues that certain strains of the *Aeromonas* genus produce guanine crystals naturally, the team decided to replicate the crystal-producing phenotype in a more liable *E. coli* laboratory strain, safer for future upscaling of guanine crystal production (Pavan et al. 2023).

### The problem: unsustainable cosmetic shimmer

Current cosmetic products on the market commonly utilize ingredients such as mica and guanine crystals to achieve a shimmering or opaque effect. However, these ingredients pose several ethical and environmental concerns. Mica is mined primarily in countries such as India, Madagascar, and China and can often involve child labor (Ghosh et al. 2023, Axim Mica Inc. 2025). Mining also entails mass deforestation and the destruction of natural habitats (Haddaway et al. 2019). Meanwhile, guanine crystals extracted from fish scales are a non-vegan product that fails to align with growing customer aversion to animal-based ingredients involving animal cruelty (Funt et al. 2017). Therefore, the team proposed an alternative product that would overcome these issues.

### The solution: guanine crystal synthesis in *E. coli* bacteria

**Figure 1.**
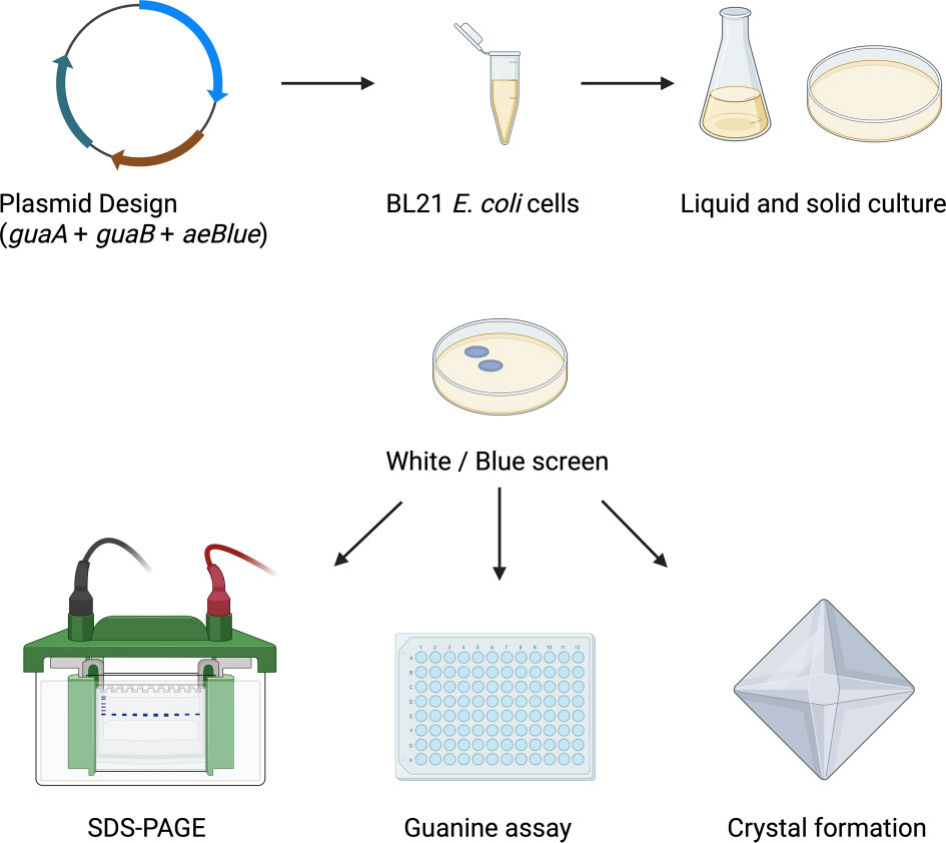
BioGlimmer project experimental design. A plasmid with the guaA-guaB BioBrick and the aeBlue expression marker was transfected into *E. coli*. Transformation was validated with the white/blue screen, guaA-guaB overexpression was measured with SDS-PAGE, guanine assays looked for signs of guanine overproduction.

BioGlimmer involved the design of two plasmids to induce excess guanine production in the laboratory workhorse *E. coli* K-12, resulting in crystal formation. The team decided to target the *guaA* and *guaB* guanine synthesis genes, as well as the guanine degradation gene *guaD. guaA* and *guaB* code for the enzymes guanosine monophosphate synthetase and inosine monophosphate synthetase, which are involved in guanine synthesis (Pavan et al. 2023). *GuaA* and *guaB*, along with the transformation marker aeBlue, were cloned into plasmids and transfected into *E. coli* to upregulate the production of guanine (Fig. 1). To ensure accumulation of guanine in the cells, the guanine deaminase gene *guaD* was targeted with a plasmid-encoded CRISPRi knockdown system. Together, these plasmids were intended to work in tandem to result in the buildup of guanine, consequently leading to crystal formation. The *guaA-guaB* overexpression plasmid under the inducible T7 promoter was successfully transformed into the *E. coli* bacteria and resulted in a detectable overexpression through SDS-PAGE of the genes of interest—*guaA* and *guaB*, therefore fulfilling the first step of producing sustainable shimmer with synthetic biology. Further steps to confirm increased guanine production involve determining actual changes in guanine concentration, followed by further rounds of metabolic engineering to ensure crystal formation.

### Importance for sustainability and synthetic biology

The BioGlimmer project adds to the development of sustainable and ethical cosmetic products through the use of synthetic biology, an approach that is still innovative to the cosmetics industry. By identifying an unsustainable component of the supply chain and applying synthetic biology principles to the low-risk bacterial strain *E. coli* K-12, which is a low risk bacterial strain, production of a shimmer product that would overcome the need for finite resources was proposed. Their project integrates the UN Sustainable Development Goals (SDGs) 8, 12, 13, and 14: promoting decent work conditions, responsible consumption and production as well as raising awareness about climate issues and protecting life under water. Therefore, BioGlimmer has illustrated the importance of promoting sustainable development through utilizing synthetic biology particularly in the beauty industry.

### The fashion and cosmetics village

In 2024, iGEM introduced a new village—Fashion and Cosmetics, in which iGEM Stockholm competed. This village was introduced in response to the growing interest in synthetic biology in the fashion and cosmetics industry, as new ideas have emerged including biopigments, protein- and cell-based materials and biofragrances. The overall winner of this village, in 2024, was iGEM IISER TVM (Indian Institute of Science Education and Research, Thiruvananthapuram). Their project utilized plastic waste to synthesize major components of sandalwood oil. Other innovative projects within this village included sustainable production of reef-safe UV filters, enzyme-based recycling of textile fibers that can be explored on the iGEM 2024 team list under the “Fashion and Cosmetics” village (iGEM Teams 2025).

## Protecting olive trees by iGEM Thessaly

The iGEM Thessaly overgraduate team tackled the environmental and economic devastation caused by the fungus *Verticillium dahliae* to Mediterranean olive trees (Papachatzis et al. 2013, Castro et al. 2020). Besides being of agricultural and economic impact, olive trees are also an important cultural asset to the region, they are central in Greek mythology and tradition, which the team noted and incorporated in their presentation. The fungal pathogen *V. dahliae* poses a significant threat not only on the local but on the global olive production, causing Verticillium wilt, a disease that kills the tree and ruins the harvests (Zhang et al. 2022). The Thessaly team used synthetic biology to offer a solution and rightfully secured multiple nominations and awards during the competition. Firstly, they were awarded with a gold medal, which showcases the great progress they made while working on this project. They were also nominated for the “Best Education” award. Education and spreading knowledge on synthetic biology is one of the core values of iGEM. The team was nominated for the ‘Best Agriculture Project’ award and won the one for the “Best New Basic Part.” The latter is worth highlighting because of the potential of the regulatory BioBricks they present acting as a base for further advancements in dsRNA production and packaging in vesicles.

### The problem: Verticillium wilt

While the team chose to focus on the olive trees because of its connection to the local community, the disease can affect other crop types. Some of which, similarly to olives, are of high economical and nutritional value such as tomatoes. It is currently impossible to assist the affected trees in recovering. They also lack resistance to this pathogen and usually do not recover after infection. These facts were affirmed by local growers, as this connection of the project to the affected people is highly valued by the iGEM ecosystem, as it is the source of creating real-world impact. Upon infection, *V. dahliae* blocks the xylem of the olive trees, hindering the transport of water and nutrients (Trapero et al. 2018). Once infection has taken place, signified by leaf discoloration and wilting. The fungus spreads by releasing microsclerotia from dying plant tissue into the soil, where it can lay dormant for as long as 20 years. Climate change accelerates its spread by creating more favorable conditions for the fungus (López-Moral et al. 2023).

### The solution: bacterial-mediated RNA interference

The ultimate goal of the team was to create a treatment solution for Verticillium wilt. Thessaly’s project was based on *Pseudomonas putida*, a plant growth-promoting rhizobacterium. The team engineered such a bacterial isolate to produce double-stranded RNA (dsRNA) in order to subsequently silence critical *V. dahliae* genes using RNA interference (RNAi). The chosen genes deemed essential for fungal survival and virulence to be silenced were *VdAAC* (energy production), *VdRGS1* (spore germination and growth regulation), and *VdTHI20* (thiamine biosynthesis). By doing so, the team aimed to inhibit the growth of the fungus without non-specific effects on other organisms. Outer membrane vesicles (OMVs) produced by the engineered *P. putida* isolate were used to effectively transfer the dsRNA. In order to guarantee that the dsRNA reached the fungal target and exerted its function, OMVs would work to shield it during delivery. The bacterial strain is intended to colonize the roots of olive trees, protecting them during crucial phases of tree growth. This method used a self-sustaining microbial culture to decrease the requirement for expensive industrial manufacture of dsRNA and OMVs. The dsRNA molecules target only specific essential genes and should, in theory, not have any toxic properties. The idea is flexible on a molecular level, making it simple to modify to treat different infections using RNA interference and OMV technology. It is a self-sufficient persistently colonizing biological system that constantly generates OMVs and dsRNA, rendering it economically relevant (Thessaly - iGEM 2024 2025).

### Importance for sustainability and synthetic biology

Thessaly’s project exemplifies how synthetic biology can improve agricultural yields while remaining sustainable. By using RNAi technology, the project avoids harmful chemical treatments and therefore supports biodiversity conservation. The use of *P. putida* as a plant growth-promoting rhizobacterium further enhances soil health, making it an enticing alternative for all olive growers compared to any other existing one. The modularity of the system means that it can be adapted to target other plant pathogens, opening a world of potential. Upon completion the project can address agricultural needs, pertinent to the UN SDGs 2, 12, and 13 related to reducing hunger, promoting sustainable agriculture, and averting climate change. It can also lead to a significant improvement of the local economy rand contribute to even further education and inspiration of young people on relevant topics. Thus, while the Dandelion project, called Tarakate, from team Marburg won this year’s agriculture prize, the Thessaly team showcased an interesting connection of a feasible project to the local community in a way that is quite characteristic and in the spirit of iGEM. The novel product or service resulting from the team's efforts can have significant real-world impact after addressing some project obstacles that still remain. Linking the gene expression observed by the proxy of GFP to a functional, beneficial outcome needs to be demonstrated. Secondly, the stability of the system in natural environments outside of the laboratory should be assessed. Finally, before pursuing commercialization, the system needs to be tested on a smaller scale to determine if it remains effective and consistent between different applications.

## Filtering heavy metals from water by iGEM KU Leuven

KU Leuven’s iGEM overgraduate project addressed two problems at once: environmentally damaging metal mining, and cleaning of metal-contaminated water. KU Leuven recognized these two problems and wondered if one could not be the answer to the other, bridged by synthetic biology. Enter KU Leuven iGEM 2024 project NeoMineX, a project that addresses the issue of heavy metal pollution in water, a pressing global environmental and health concern. This pollution, primarily caused by industrial processes like mining, electronics manufacturing, and wastewater discharge, leads to toxic bioaccumulation in ecosystems and poses severe risks to human health (Singh et al. 2024). As the world grapples with environmental degradation and resource scarcity, finding sustainable efficient methods for metal recovery and pollution remediation is crucial for a cleaner future.

### The problem: heavy metal water pollution

Heavy metals, such as cadmium, copper, zinc, lead, and nickel are essential in modern industries, but their extraction and disposal often harms the environment. Industrial activities discharge these metals into water systems, contaminating freshwater sources (Aziz et al. 2023). This not only disrupts ecosystems but also presents long-term risks to biodiversity and human populations (Tchounwou et al. 2012). Current methods for metal recovery and wastewater treatment, such as chemical precipitation and ion exchange, are energy-intensive, inefficient, and generate secondary pollutants.

### The solution: metal-binding proteins

The KU Leuven team proposed a synthetic biology-based solution that combines bacterial engineering with innovative biotechnological tools. They genetically modified *E. coli* bacteria to be capable of selectively binding heavy metals from wastewater. First, the team used AlphaFold to determine the structure of the proteins Csp1 binding to copper and SmtB binding to cadmium and zinc. These metal-binding protein (MBP) genes were cloned into *E. coli* (Permyakov 2021). Using metal ion gradient assays, the transfected strains showed a higher tolerance for metal ions in the environment, indicating that the bacteria were able to absorb and neutralize the ions using the MBPs. Ultimately, the team intends for the bacteria to be put in contact directly with wastewater to recover heavy metals from the ecosystem. By then separating these genetically engineered *E. coli* from the wastewater, these metals would be able to be recovered in concentrated form, by separating the *E. coli* from the wastewater and either burning or treating with acid to recover the metals. The NeoMineX system provides a dual benefit: it cleans wastewater while recovering valuable metals, contributing to a circular economy. This solution addresses both environmental and economic aspects of the problem by reducing pollution and reclaiming resources (KULeuven - iGEM 2024 2025).

### Importance for sustainability and synthetic Biology

The NeoMineX project underscores the transformative potential of synthetic biology in sustainable practices: by recovering and recycling heavy metals, this solution reduces reliance on environmentally damaging mining practices, promoting resource efficiency and sustainability. The biological approach also minimizes secondary waste and energy consumption, presenting a cleaner alternative to traditional chemical methods. The solution is also scalable: engineered bacteria are relatively easy to produce and scale, making this approach adaptable to various industrial settings. In general, the project showcases the power of synthetic biology to create highly specific and efficient tools for environmental applications. The use of biosensors exemplifies integration with digital tools for smarter, data-driven resource management. The implications of KU Leuven’s work extend beyond water purification. As industries increasingly prioritize sustainability, solutions like NeoMineX demonstrate how synthetic biology can address global challenges. The project highlights the potential for microbiological systems to function as eco-friendly tools for tackling environmental pollution while simultaneously contributing to economic resilience through resource recovery. Additionally, NeoMineX fosters collaboration between synthetic biology, environmental science, and engineering, paving the way for interdisciplinary innovations. This aligns with global sustainability goals, including responsible consumption, clean water access, and climate action. In conclusion, the KU Leuven iGEM 2024 project is moving bioremediation one step closer by merging advanced microbiological techniques with sustainability principles, NeoMineX provides a replicable model for solving environmental problems in a resource-efficient and economically viable manner. And though there is still much work to be done to implement such a project in practice, the results are very encouraging and inspiring.

## Filtering endocrine-disrupting chemicals from breast milk by iGEM Copenhagen

The iGEM University of Copenhagen 2024 project addressed the growing problem of endocrine-disrupting chemicals (EDCs) released into the environment from industrial processes. The Copenhagen team was nominated for the Best Food & Nutrition Project, Undergrad & Overgrad, as well as for the Best Part Collection, Overgrad; Best Model, Overgrad; and Best Presentation, Overgrad. Infants are especially vulnerable to this type of chemical and can even be exposed through breast milk (Serreau et al. 2024). However, breastfeeding is highly beneficial for the infants promoting enhanced immunity and cognitive development, as well as increasing the bonds between parent and child (Modak et al. 2023). These benefits can be lost with the increasing presence of pollutants. The objective of the Copenhagen team was to develop a dual system that is able to detect and detoxify contaminants in breast milk with synthetic biology.

### The problem: endocrine-disrupting chemicals

EDCs are chemicals used in consumer products, such as bisphenol A, common to plastics, that also can be harmful to humans and the environment. By being chemically compatible with human endocrine systems, EDCs can interfere with hormone-dependent signaling pathways related to fetal development, brain homeostasis, metabolism, and reproduction (Dwivedi et al. 2023). Infants are exposed to these chemicals through breast milk, as many EDCs accumulate in adipose tissues due to their chemical nature and this includes breast milk. The primary liver enzymes, in charge of detoxification, are undeveloped in infants, which puts them at a higher risk when exposed to EDCs (Predieri et al. 2022). This exposure has been correlated with several health issues, including cognitive disorders, reproductive issues such as early puberty and reduced fertility, and other potential lifelong health consequences (Braun 2017). Current solutions to the problem involve stopping breastfeeding, which sacrifices the benefits of it (Modak et al. 2023).

### The solution: SUPERMOM combines detection and detoxification

The solution developed by the University of Copenhagen iGEM team is called SUPERMOM (UCopenhagen - iGEM 2024 2025). The project is designed as a two-part system that provides detection and detoxification of contaminants in breast milk. Its development was inspired by the natural detoxification process of the liver, a process that is present (at least in part) also in lower eukaryotes such as *Saccharomyces cerevisiae* (Grimm et al. 2015, Shmarakov et al. 2017). SUPERMOM operates through a specific engineered yeast-based biosensor that specifically detects a harmful substance. Upon ligand perception, the biosensor will bind and initiate expression of the detoxification system to neutralize the contaminant. For a proof-of-principle experimental set-up, the EDC pollutants bisphenol A (BPA), dioxin and polychlorinated biphenyl (PCB) were selected. IBA is detected by the estrogen receptor alpha and estrogen-related receptor gamma, while the detection of dioxin and PCB is carried out by the aryl hydrocarbon receptor. Phase I of the detoxification step is subsequently initiated by the enzymes p450 reductase and cytochrome P450, which will modify contaminants, detoxifying them or preparing them for phase II, which is not present in *S. cerevisiae*. carry out In the second detoxification phase enzymes of the Uridine 5′-diphospho-glucuronosyltransferase (UGT) family detoxify contaminants by transferring glucuronic acid from UDP-a-D-glucoronate onto the contaminant. To be created from UDP-a-D-glucose, UDP-glucose dehydrogenase was successfully coexpressed with UGT to carry out the transfer reaction. Strikingly, SUPERMOM achieved all their main objectives. With its initial focus on successfully detecting and detoxifying BPA, dioxin, and PCB, there is the realistic potential to expand the targets to a wider range of diverse EDC contaminants.

### Importance for sustainability and synthetic biology

The Copenhagen iGEM 2024 team project exemplified the use of synthetic biology to reduce the impact of the unsustainable growing industry releasing harmful chemicals such as EDCs. Their method of purifying breast milk by mimicking the liver detoxification system aids to nurture future generations and minimizes the risk of being exposed to these contaminants. Through their work, the team showed how synthetic biology tools can be used to address environmental problems affecting millions of infants and a possibility of expansion into a wider range of contaminants. Their project also integrates UN SDGs 2, 3, and 10. Zero hunger, by ensuring safe breastfeeding, good health and well-being by the prevention of complications due to EDC exposure and reducing inequalities within and among countries. The project demonstrates the potential use of microbes to eliminate contaminants to challenge the growing industries and economies to look for sustainable solutions.

## Conclusions: the impact of iGEM on synthetic and sustainable microbiology

Microbial approaches present a promising alternative to address challenges in sustainability. Synthetic biology relies on engineered microorganisms to unlock new abilities for useful purposes. The iGEM vision is a world where synthetic biology provides sustainable, effective, and safe alternatives and solutions. At the 2024 iGEM Grand Jamboree, the 14 villages within the three macro-categories of environment, healthcare, and technological advancements, reflected the major area of expertise of more than four hundred research projects. Here, four 2024 iGEM research projects are described to explore the potential of synthetic biology applied to microbes as a sustainable solution to current global challenges. In particular, engineered microbes are shown to function as environmental safeguards, thereby addressing challenges in the agricultural sector, as well as promoting responsible consumption and production, and contributing to health advances.

The annual iGEM Grand Jamboree showcases cutting-edge synthetic biology proposals with the potential to shape the future. In light of this, it is clear that actively training synthetic biologists in how to meet societal needs is a key element of the iGEM competition. In fact, the competition requires its participants to place a strong emphasis on the performance of human practice activities. The aim is to encourage students to reflect on the societal, ethical, environmental, safety, and security impact of their research projects. This includes promoting responsiveness, responsibility, and education. The rationale behind this approach is that only by connecting scientific research to real-world contexts is it possible to gain insights into the practical needs of society and the environment, and subsequently apply synthetic biology in such ways that will benefit both (iGEM Responsibility 2025).

iGEM thus recognizes the potential contribution of synthetic biology research to the achievement of SDGs. By encouraging high school and university students to adopt a proactive approach to responsible innovation, iGEM ensures that the potential applications and implications of all the research proposals are taken into account in a safe and sustainable vision. In this context, all the proposed research projects have been carefully considered. This is of paramount importance, especially when considering the potential danger of engineered microbes if released into the environment. Overall, the entire iGEM journey is an enriching experience for students, both from a research and a personal point of view. The encouraged collaboration between iGEM teams as well as the international meetings provide a great opportunity for networking. In addition, the possibility to explore other teams' ideas, as well as the required discussions with a panel of experts, helps to develop a critical research mindset that will make the difference in the next generation of researchers devoted to achieving sustainable goals.

## Data Availability

All relevant data are contained within this report.
